# The effect of in-lab polysomnography and home sleep polygraphy on sleep position

**DOI:** 10.1007/s11325-020-02099-w

**Published:** 2020-05-16

**Authors:** Wojciech Kukwa, Ewa Migacz, Tomasz Lis, Stacey L. Ishman

**Affiliations:** 1 Department of Otorhinolaryngology, Faculty of Dental Medicine, Medical University of Warsaw, 19/25 Stepinska Street, 00-739 Warsaw, Poland; 2 Department of Pediatric ENT, Medical University of Warsaw, Warsaw, Poland; 3 Division of Otolaryngology – Head and Neck Surgery, Cincinnati Children’s Hospital Medical Center, Cincinnati, OH USA; 4 Division of Pulmonary Medicine, Cincinnati Children’s Hospital Medical Center, Cincinnati, OH USA; 5 Department of Otolaryngology – Head and Neck Surgery, University of Cincinnati College of Medicine, Cincinnati, OH USA

**Keywords:** Obstructive sleep apnea, Sleep position, Positional sleep apnea, Polysomnography, Home sleep apnea test, Sleep study

## Abstract

**Purpose:**

Little is known regarding the influence of in-laboratory polysomnography (PSG) equipment on sleep position, especially on the prevalence of supine positioning, which in many cases may lead to a more severe sleep apnea diagnosis. The aim of this study was to assess the percentage of supine sleep during an in-laboratory PSG compared to that seen during a home sleep apnea test (HSAT).

**Methods:**

This was a retrospective cohort study comparing in-laboratory PSG and HSAT using a peripheral arterial tone (PAT) technology device.

**Results:**

Of 445 PSG and 416 HSAT studies analyzed, there was no significant difference in the proportion of supine sleep time between PSG (44%) and HSAT (45%, *p* = 0.53). Analysis of the differences in sleep position (supine versus non-supine), analyzed by sex, BMI (≥ 30 kg/m2 versus < 30 kg/m2), and age (≥ 60 years versus < 60 years), was significant only for women, who had more supine sleep during HSAT at 61 ± 24% than during PSG at 45 ± 26% (*p* < 0.001).

**Conclusion:**

Overall there was no difference in the percentage of supine sleep when comparing in-laboratory PSG to HSAT. However, women had more supine sleep with HSAT than with PSG.

## Introduction

Polysomnography (PSG) is the widely accepted gold standard to diagnose obstructive sleep apnea [1] in both children and adults [2–4]. It is a comprehensive sleep evaluation based on numerous physiologic parameters, including oxygen saturation, brain electroencephalographic activity, eye movements, muscle activity, heart rate and rhythm, and respiratory function. Measurement of these parameters is carried out using monitors including surface electrodes attached to a PSG headbox, which typically is placed on the patient’s chest. When undergoing PSG, patients spend a night in a sleep laboratory while continuously supervised by a sleep technician.

The origins of PSG date back to the 1950s, when scientists started performing full-night recordings of physiological signals during sleep. Beginning in the early 1960s, sleep researchers started to apply this technology to study sleep pathology [5]. Although PSG is the gold standard for the diagnosis of OSA, it has many limitations including high cost, limited availability, and performance in an artificial sleep setting in the sleep laboratory [6]. Additionally, as demonstrated in 1985 by Cartwright et al., some patients report feeling constrained during in-laboratory PSG due to the presence of numerous leads and monitors resulting in them spending more time in the supine position than they would have during a typical night at home [7]. In light of the fact that OSA is often more severe in the supine position, the assessment for OSA in the sleep laboratory may be misleading and lead to overdiagnosis of patients with positional OSA which can result in inappropriate therapy. A 1996 study by Metersky et al. (*n* = 12) measured the amount of supine sleep during an in-laboratory nighttime PSG and compared it to an in-laboratory night without any sleep study equipment attached to the patients [8]. This author found that the mean percentage of supine sleep was 49% in the sleep lab which was 56% greater than was seen during the non-PSG night.

In light of these findings and the fact that there are no large studies that analyze the potential effect of the in-laboratory PSG setup on sleep position, the aim of this study was to retrospectively assess the rate of supine sleep during a traditional in-laboratory PSG and to compare it to the rate of supine sleep seen during a home sleep apnea test (HSAT).

## Methods

### Participants

This was a retrospective cohort study from January 2015 to June 2018 that included consecutive sleep studies for adult patients who underwent in-laboratory PSG or HSAT using PAT technology devices. The inclusion criteria specified patients 18 years or older with a clinical suspicion of OSA who did not have any previous history of treatment for OSA. We excluded patients with a PSG-measured total sleep time (TST) or a PAT-measured true sleep time (pTST) of less than 300 min.

### Polysomnography

The in-laboratory PSG was conducted using a Nox A1 PSG System (Nox Medical, Reykjavik, Iceland). The following signals were recorded simultaneously: 6-channel electroencephalography (leads: F3A2, F4A1, C3A2, C4A1, O1A2, O2A1), left and right electrooculography (LOC, ROC), and submental electromyography (3 unipolar leads). In addition, nasal airflow pressure was acquired with a nasal cannula, respiratory effort was assessed with thoracic and abdominal inductive plethysmography, and snoring was recorded using a built-in microphone. Body position and activity were measured with the internal 3-axis, ± 2-g accelerometer with 10 Hz sampling frequency. The device was attached with the clips snapped to the patient’s shirt on the thorax with dimensions of 82 mm W × 63 mm H × 21 mm D and a mass of 163 g. Pulse, oximetry, and plethysmography were measured with a Nonin 3150 WristOx2™ wireless oximeter (Nonin Medical, Plymouth, MN, USA). Subjects were supervised by a polysomnographic technician.

Sleep staging and respiratory scoring were interpreted by a sleep physician using the standard criteria defined by the American Academy of Sleep Medicine [4]. The apnea-hypopnea index was defined as the combined number of apneas and hypopneas that occurred per hour of sleep. Hypopnea was defined as a 30% decrease in airflow for at least 10 s, followed by a 4% decrease in saturation. Respiratory event-related arousals (RERAs) were not scored by the sleep laboratory.

### Home sleep apnea test

The home-based sleep study was performed using the WatchPAT™200 (Itamar Medical Ltd., Caesarea, Israel) portable sleep apnea diagnostic system, which tracks peripheral arterial tonometry, oximetry, heart rate, actigraphy, body position, and snoring.

The body position sensor used a 3-axis accelerometer that produced a signal directly proportional to the patient’s sleeping posture (supine, prone, right side, left side, and sitting) with a sampling frequency of 100 Hz. The sensor was attached with an adhesive sticker on the patient’s chest right under the sternal notch. The sensor was 32 mm in diameter with a mass of 12 g.

A sleep technician instructed each patient on how to affix the sensors to ensure proper placement of a device on the same day that the study was carried out. The study was automatically analyzed using Itamar’s software which uses a 4% desaturation criteria.

### Statistical analysis

Because the data was not normally distributed, the Mann-Whitney test was used to compare variables between PSG and HSAT groups. Linear regression models were applied to predict if any factor (as sex, age, BMI, or study type) influences supine position sleep. Statistical analysis was performed using IBM SPSS Statistics for Windows, version 20 (IBM Corp., Armonk, N.Y., USA).

## Results

There were 445 PSG and 416 HSAT studies analyzed. The mean age of the HSAT cohort was younger at 48.7 ± 13.8 years than for the PSG group at 54.1 ± 13.3 years (*p* < 0.001). Women constituted 31% (138) of patients undergoing PSG and 12% (50) of those undergoing HSAT (*p* < 0.001). The patients undergoing HSAT also had a lower BMI (29.7 ± 5.6 kg/m^2^) than those patients undergoing PSG (31.1 ± 5.9 kg/m^2^, *p* < 0.001).

As seen in Table 1, there was no significant difference in the overall proportion of sleep time spent in the supine position in the cohorts undergoing PSG (44.1%) versus those who underwent HSAT (44.6%, *p* = 0.53) despite longer TST for the HSAT group than the PSG group (*p* < 0.001). The overall AHI for the PSG group (23.1 ± 41.4 events/h) was higher than the pAHI (17.5 ± 22.6 events/h) for the HSAT group (*p* = 0.009).

**Table 1:** Baseline demographic data for adults undergoing either HSAT or PSG testing to assess for obstructive sleep apnea

	Sleep test	*n*	Mean (SD)		*p* value*
Age (years)	HSAT	416	48.7	(13.8)	*< 0.001*
	PSG	445	54.1	(13.3)	
BMI (kg/m^2^)	HSAT	414	29.7	(5.6)	*< 0.001*
	PSG	435	31.1	(5.9)	
Sleep time (minutes)	HSAT	416	405.2	(57.4)	*< 0.001*
	PSG	445	379.7	(31.1)	
Supine (%)	HSAT	416	44.6	(25.1)	0.528
	PSG	445	44.1	(27.2)	
pAHI AHI	HSAT	416	17.5	(22.6)	*0.009*
	PSG	445	23.1	(41.4)	

HSAT, home sleep apnea testing; PSG, polysomnography; SD, standard deviation; BMI, body mass index; n, number; pAHI, PAT (peripheral arterial tone) apnea hypopnea index; AHI, apnea hypopnea index; *, Mann-Whitney test

The significance was for *p* < 0.05

Table 2 shows the differences in percentage of total sleep in the supine position subgrouped by sex, BMI (≥ 30 kg/m^2^ versus < 30 kg/m^2^), and age (≥ 60 years versus < 60 years). The only significant finding was that women had more supine sleep during HSAT (60.8 ± 23.9%) than they did during PSG (45.4 ± 25.8%, *p* < 0.001). In the PSG group, the mean supine AHI was 46.87 ± 30.23 events/h compared with 23.14 ± 41.37 non-supine. For the HSAT group, the mean supine AHI was 29.58 ± 26.56 events/h compared with 17.47 ± 22.59 non-supine.

**Table 2:** HSAT and PSG percentage of supine sleep for adults undergoing evaluation for obstructive sleep apnea subgrouped by sex, body mass index (BMI), and age

	Sleep position	Sleep study	*n*	Mean	(SD)	*p* value*
Sex						
Female						
	Supine (%)	HSAT	50	60.8	23.9	*< 0.001*
		PSG	138	45.4	25.8	
Male						
	Supine (%)	HSAT	366	42.4	24.5	0.943
		PSG	307	43.5	27.8	
BMI						
< 30 kg/m^2^						
	Supine (%)	HSAT	238	46.5	25.5	0.863
		PSG	216	46.2	26.8	
≥ 30 kg/m^2^						
	Supine (%)	HSAT	176	41.9	24.6	0.442
		PSG	219	41.2	27.1	
Age, year						
< 60						
	Supine (%)	HSAT	308	45.4	24.3	0.679
		PSG	260	46.8	26.4	
≥ 60						
	Supine (%)	HSAT	108	42.4	27.4	0.451
		PSG	185	40.3	27.8	

HSAT, home sleep apnea testing; PSG, polysomnography; SD, standard deviation; n, number; kg, kilograms; m, meter; BMI, body mass index; pAHI, PAT (peripheral arterial tone) apnea hypopnea index; AHI, apnea hypopnea index; *, Mann-Whitney test

differences are signifficant at *p* < 0.05

Regression analysis of study type as a predictor of supine sleep, when controlled for sex, age, and BMI, showed that (1) women spent 8.1% more sleep time supine than did men, (2) each increase in year of age was associated with a 0.2% decrease in total sleep time in the supine position, (3) each increase of 1 in the BMI score decreased total sleep time in the supine position by 0.5%, and (4) the study type did not significantly influence the likelihood of supine sleep.

## Discussion

Overall, we found no difference in the percentage of supine sleep when comparing patients who underwent a traditional in-laboratory PSG to those assessed with a HSAT despite the fact that those undergoing HSAT were younger, had a slightly lower BMI, and had longer sleep time. Moreover, the supine AHI was higher than the non-supine AHI with both the HSAT and PSG testing. Subgroup analysis revealed that women had significantly more supine sleep with HSAT than was seen in the sleep laboratory; however, there were no differences noted for men, or when assessing this cohort by BMI or age. Literature review revealed only one mention of a percent of supine sleep for men and women, separately [9], showing a slightly higher value for women (40.5% (± 18.6)) than men (35.1% (± 18.2)).

In 1985, Cartwright et al. wrote that “Patients sometimes report in the morning that they spent more time in this (supine) position during their laboratory evaluation than they typically do at home. Sleep disorders centers need to recognize the contribution of body position to their estimation of sleep apnea severity and therefore instruct patients to occupy their usual sleep postures if possible” [7]. This is a significant concern given that many OSA patients have upper airway obstruction that is most prominent while sleeping supine, known as positional OSA (POSA). There are currently two types of POSA. The first is supine predominant obstructive sleep apnea (spOSA) where the whole-night AHI is ≥5 events/h and the supine AHI is greater than twice the non-supine AHI; this is reported to occur in up to 60% of patients who present to sleep clinics [10, 11]. The second type, which occurs in up to 30%, is supine isolated obstructive sleep apnea (siOSA) where the whole-night AHI is ≥ 5 events/h and the supine AHI is greater than twice the non-supine AHI but the non-supine AHI does not exceed 5 events/h [12–14].

A 2014 review article by Joosten et al. concluded that “the amount of time spent in supine sleep in unselected general population is not known” [12]. Similarly, Sorscher et al. in 2018 identified the need for research regarding body position during sleep with respect to normative data as well as night to night variability [15].

Table 3 includes the rates of supine sleep previously reported in the literature. Metersky et al. reported that 9 out of 12 patients spent 56% more time in the supine position during PSG night than during subsequent nights in the laboratory when not connected to the monitoring leads [8]. Our results did not confirm this but instead showed that the percentage of supine sleep was similar during multi-channel PSG and HSAT. We can speculate that the PSG used by Metersky et al. in the 1990s’ was more “uncomfortable” than the PSG used in our study and may have had greater impact on the sleep position. Similarly, a large-scale retrospective study (*n* = 168) by Vonk et al. analyzed the rate of supine sleep during in-lab PSG and compared it to the home sleep during the inactive (diagnostic) phase of sleep position trainer (SPT) [16]. While they found that there was a significant difference in the percent of supine sleep between PSG (43.1%) and SPT night (28.6%), all participants had positional OSA, which could have biased the results. Additionally, the authors informed all patients to avoid supine sleep before the SPT nights.

**Table 3:** Comparison of previously published studies which report data on the percentage of supine sleep during a sleep study

Study	Number of patients	Sleep test type	% of supine sleep
Sunnergren et al.	189	HSAT (Embletta)	39
Campbell and Neill	30	Home PSG (Compumedics Siesta) PSG	41.4 36
Sorscher et al.	49	HSAT (ApneaTrak, Cadwell)	41.2
Yin et al.	90	HSAT (Stardust II, Respironics) PSG	51.3 62.7
Metersky et al.	12	PSG	49

HSAT, home sleep apnea testing; PSG, polysomnography

A study by Skarpsno assessed multi-night actigraphic recordings of 664 adults recruited from the general population and reported that 37.5% of total time in bed was spent supine [9]. Similar proportions of supine sleep were reported in studies by Sunnergren et al. (39%), Campbell and Neill (36–41%), and Sorscher et al. (41%), while Yin et al. reported a higher proportion of supine sleep in his cohort during both in-laboratory (62%) and at-home PSG (51%) [15, 17–19]. Our findings were similar to the preponderance of studies with supine sleep seen 44% of the time for both the in-laboratory PSG and HSAT cohorts.

Limitations of this study include its retrospective nature and the selection bias implicit in using two separate groups for the comparison. While this is a pragmatic approach, ideally future studies would compare the influence of in-laboratory versus HSAT devices on sleep positions in a single cohort. In addition, while there were differences in the mean age and BMI between these 2 cohorts, subgroup analysis did not reveal any significant differences in sleep position based on these variables. Lastly, our data was collected from a single night study and thus cannot provide information about night-to-night variability of body position during sleep.

## Conclusion

We found that patients slept supine for 44% of TST in both the in-laboratory PSG and HSAT cohorts, despite differences in age and BMI between the two groups. While there are concerns that in-laboratory testing may significantly influence body position during sleep, this was not the case in our study. Women did have more supine sleep when tested in the home setting, but no differences were seen for men or in the overall cohort.
